# Evaluation of the Preservation and Digestion of Seal Meat Processed with Heating and Antioxidant Seal Meat Hydrolysates

**DOI:** 10.3390/md20030204

**Published:** 2022-03-10

**Authors:** Yi Zhang, Lea Spitzer, Xin Rui, Susana C. M. Fernandes, Romy Vaugeois, Benjamin K. Simpson

**Affiliations:** 1 Department of Food and Science and Agricultural Chemistry, McGill University, Ste-Anne-de-Bellevue, QC H9X 3V9, Canada; 2 E2S UPPA, CNRS, IPREM, Université de Pau et des Pays de l’Adour, 64000 Pau, France; lea.spitzer@univ-pau.fr (L.S.); susana.fernandes@univ-pau.fr (S.C.M.F.); 3 College of Food Science and Technology, Nanjing Agricultural University, Nanjing 210095, China; ruix@njau.edu.cn; 4 Les Entreprises SeaDNA Canada Inc., Sainte-Brigitte-de-Laval, QC G0A 3K0, Canada; sales@seadna.ca

**Keywords:** seal meat, harp seal, hydrolysates, digestion, bioprocessing

## Abstract

Seal meat is of high nutritive value but is not highly exploited for human food due to ethical issues, undesirable flavors, and loss of nutrients during the processing/cooking step. In this work, commercially available processed seal meat was treated with its hydrolysates as preservatives with the aim of improving nutrient bioavailability. The contents of the nutrients were analyzed after digestion using a simulated dynamic digestion model, and the effects of different processing conditions, i.e., low-temperature processing and storage (25 °C) and high-temperature cooking (100 °C), of seal meat were investigated. Hydrolysates with antioxidant activity decreased the amounts of the less desirable Fe^3+^ ions in the seal meat digests. After treatment with hydrolysates at room temperature, a much higher total Fe content of 781.99 mg/kg was observed compared to other treatment conditions. The release of amino acids increased with temperature and was 520.54 mg/g for the hydrolysate-treated sample versus 413.12 mg/g for the control seal meat sample treated in buffer. Overall, this study provides useful data on the potential use of seal meat as a food product with high nutritive value and seal meat hydrolysates with antioxidant activity as preservatives to control oxidation in food.

## 1. Introduction

The harp seal (*Pagophilus groenlandicus*) is a common seal species found in the Atlantic Ocean, particularly in waters that border Canada [1]. The harp seal population has increased considerably over the past decades, reaching ca. 7.6 million species in 2019 [2], which is creating a fish stock crisis due to the large amounts of fish consumed annually by the seals [3]. Responsible harvesting is one way to control the seal population and maintain sustainable fisheries and the ocean economy [4]. Nonetheless, the underutilization of seal meat hinders the commercial progress of seal meat products in local and international markets. Seal meat is still predominantly considered a byproduct or waste from seal oil extraction [5], as well as fur and leather fabrication, with the bulk of this byproduct or waste ending up as fertilizer, animal feed, or silage [6,7].

As food, seal meat is commercially processed into different cuts, such as loins, flippers, and trims, and is consumed in a number of countries, including Canada, Greenland, Japan, Norway, Iceland, and Siberia [8]. Although seal meat has great potential as a component of the human diet, its consumption and the variety of its products are currently limited. High-temperature processing is still a commonly used treatment method for seal meat products, including sausage and jerky. Seal meat is considered lean meat and is a potentially interesting food source due to its well-balanced amino acid composition [9]; high protein content (around 28 g per 100 g serving) [1,6]; low fat level (3%), which is mostly composed of monounsaturated and polyunsaturated fatty acids; and high amounts of vitamins and minerals, especially vitamin B_12_, iron (Fe), magnesium (Mg), and zinc (Zn) [10]. Taking Fe as an example, the consumption of a 10 g serving of seal meat provides ca. 25% of the recommended daily intake of iron, which is about 17 times higher than that obtained from beef, pork, poultry, or fish [11]. Because heme ion (Fe^2+^) is the form that is readily absorbed in the human gastrointestinal tract [12], it is necessary to investigate the ratio between Fe^2+^ and Fe^3+^ obtained from seal meat after digestion. The conventional preservation methods for fresh seal meat cuts include freezing, vacuum packaging, and canning, and long-term storage can cause the gradual oxidation of Fe^2+^ and nutrient loss. The processing of seal meat at high temperatures (cooking) can lead to the oxidation of the preferred Fe^2+^ form to the less desirable Fe^3+^ form [1]. Recently, the production of seal meat hydrolysates with antioxidant activities using an enzymatic hydrolysis approach was reported [13], which showed their potential to be used as natural food preservatives for fresh seal meat. Accordingly, we hypothesized that a pre-treatment step in the preparation of fresh seal meat using seal meat hydrolysates with antioxidant activity may decrease the oxidation of seal meat to ensure high nutrition value. Thus far, no studies have been reported on the implementation of a bioprocessing method (e.g., treatment with protein hydrolysates) combined with traditional high-temperature processing for seal meat. Reports on the bioavailability and nutrient values of seal meat after digestion are also scarce. In this study, new bioprocessing strategies using seal meat hydrolysates to treat commercially available frozen seal meat products were investigated to determine: (i) the antioxidant effect of seal meat hydrolysates as potential preservative agents for seal meat and (ii) the effect of processing methods with low- and high-temperature treatments and hydrolysate treatment on the digestibility and nutrition intake (amino acids and minerals) of seal meat.

## 2. Results and Discussion

### 2.1. Seal Meat Hydrolysates as Food Preservatives with Antioxidant Activity

**Figure 1:**
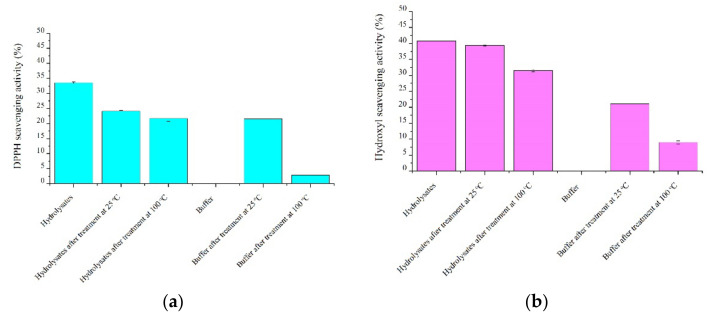
(**a**) DPPH free radical scavenging activity and (**b**) hydroxyl radical scavenging activity of the hydrolysates and buffer solutions collected after treatments at 25 °C and 100 °C.

Reactive oxygen species (ROS) and free radicals are usually created during food processing, storage, and cooking. Antioxidant reagents, such as antioxidant hydrolysates, can react with ROS to extend the shelf life of food products. The antioxidant properties were analyzed for “Hydrolysates 25”, “Hydrolysates 100”, “Buffer 25”, and “Buffer 100”, as well as “Buffer” and “Hydrolysates”. DPPH free radical scavenging activities, as shown in Figure 1a, were 33.5% for the original hydrolysates, 24.1% for the hydrolysates after being used to treat the seal meat at 25 °C, and 21.7% after treatment at 100 °C. As shown in Figure 1a, the buffer solution collected after being used for the treatment of the seal meat at 25 °C showed DPPH scavenging activity of 21.6%, while for the treatment at 100 °C, DPPH scavenging activity in the buffer solution amounted to 2.9%. The observed DPPH activity for the buffer treatments suggests that it is possible to generate hydrolysates with antioxidant activity from seal meat treated at 25 °C and 100 °C. On the other hand, the observed reduction of DPPH for the hydrolysate treatments suggests that the hydrolysates can act as antioxidant agents to scavenge ROS in the seal meat during low-temperature processing or storage (at 25 °C) and high-temperature processing (at 100 °C). The hydroxyl free radical scavenging activity, as shown in Figure 1b, followed a similar trend, suggesting that the hydrolysates have higher antioxidant activity. Hence, the treatments with antioxidant hydrolysates were more effective in improving seal meat quality, presumably because the hydrolysates act as inhibitors of lipid oxidation and hydrophilic ROS, the major causes of the reduction in the quality of meat [14]. Indeed, previous studies have reported the use of antioxidant protein hydrolysates and/or antioxidant peptides as food preservatives or food additives from various sources, such as fish, soya, eggs, and others [15,16]. Currently, only a few protein hydrolysates and peptides are accepted as food preservatives/additives, including nisin [16] and pediocin [17]; however, their use requires specific conditions [18], as peptides generally have high sensitivity to external environments [19]. Thus, further studies on protein hydrolysates, including gastrointestinal stability and non-toxicity, are necessary prior to their application as food preservatives or additives [20].

It is also worth noting that processing at an elevated temperature (100 °C) affected the antioxidant properties of hydrolysates and buffer solutions compared to processing at 25 °C. The antioxidant properties of protein hydrolysates and peptides depend not only on their amino acid compositions but also on their structural features [21,22], which are unstable at high-temperature conditions. Thus, the antioxidant effect of the hydrolysates on seal meat is expected to be relatively low at high temperatures. To assess the use of seal meat hydrolysates as antioxidant agents in foods, further investigations on treated seal meat to determine parameters such as oxidation degree, antimicrobial activity, cholesterol-lowering ability, and allergenic potential before and after treatment using seal meat hydrolysates are necessary [15,23].

### 2.2. Amino Acid Profiles of Treated Seal Meats after Digestion

After treatments with the hydrolysates or buffer at 25 °C or 100 °C, seal meats were collected, freeze-dried, and digested via the dynamic rat digestion model, and the liquid digests after stomach and intestine digestions were collected, as described in Section 3.4, for further characterization.

**Figure 2:**
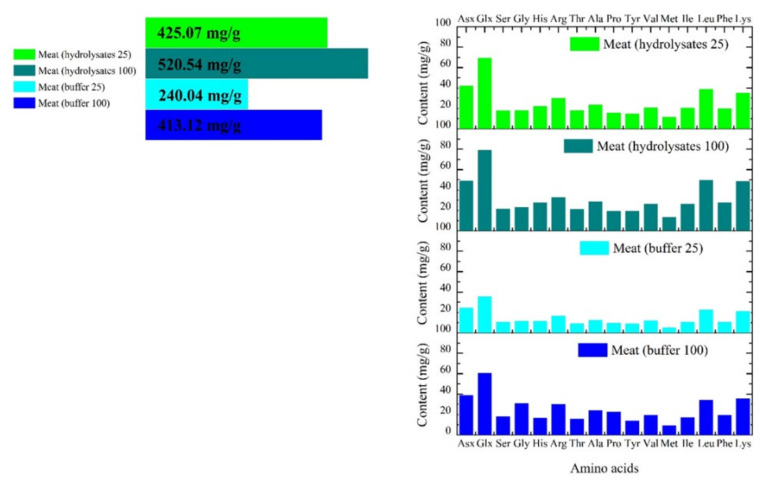
Total amino acid contents and their profiles (mg/g) of the intestinal digests of seal meat treated with hydrolysates or buffer at 25 °C and 100 °C, respectively.

For the amino acid analysis, only the intestinal digests were considered, as amino acids are mainly absorbed in this portion of the GIT [24]. Figure 2 shows the amino acid profiles, showing the total amino acid contents of differently treated meat digests. A general increase in the content of amino acids was observed at higher temperatures, from 240.04 mg/g (25 °C) to 413.12 mg/g (100 °C) for the seal meat treated with buffer and from 425.07 mg/g (25 °C) to 520.54 mg/g (100 °C) for the seal meat treated with hydrolysates. Amino acids with high contents in the four samples were Glx (Glu + Gln), Asx (Asp + Asn), Leu, and Lys, with the highest amounts in the digests from seal meat after the hydrolysate treatment at 100 °C (79.56, 49.60, 50.17, and 49.16 mg/g). A higher temperature increases protein denaturation and the breakdown of the seal meat network, which facilitates the release of polypeptides and peptides into the digestive milieu in the GI model, where enzymes digest them into amino acids. Thus, temperature-induced protein denaturation at the higher temperature resulted in a higher amino acid content in the 100 °C treated samples than in the 25 °C treated samples. Thus, the high-temperature processing of seal meat effectively improved the bioavailability and nutritive value of the seal meat regarding the content of amino acids. Furthermore, treatments using the hydrolysates resulted in a higher amino acid content than the control treated in buffer.

The amino acid composition results of the four samples showed similar trends. Table 1 summarizes the number of each amino acid per 1000 amino acids. There were relatively high numbers observed for the amino acids Glx, Asx, Gly, Leu, Ala, and Lys. Among them, Leu and Lys are considered essential amino acids. In general, seal meat has a high amount of Lys [10], an essential amino acid vital for the immune system and protein and hormone synthesis [25,26]. In this study, the ratio between essential amino acids (EAAs) and non-essential amino acids (NEAAs), i.e., EAA/NEAA, increased from 0.59 in the buffer treatments at 100 °C to 0.770 in the hydrolysate treatments at 100 °C. EAAs are vital and need to be supplied by a suitable diet, since they cannot be produced in significant amounts in the human body [27]. The EAA/NEAA ratio, therefore, represents an important indicator of the nutritive value of food. Comparable to other EAA-rich food protein sources, such as goat (EAA/NEAA = 0.88 [28]) and swordfish (EAA/NEAA = 0.73–0.92 [29]), seal meat treated by hydrolysates at high temperature could also be considered a good source of EAAs. Thus, processing seal meat with hydrolysates at high temperatures was shown to be a promising strategy to improve the potential nutritive value of seal meat in terms of essential amino acid production.

### 2.3. Mineral Profiles of Treated Seal Meats after Digestion

**Figure 3:**
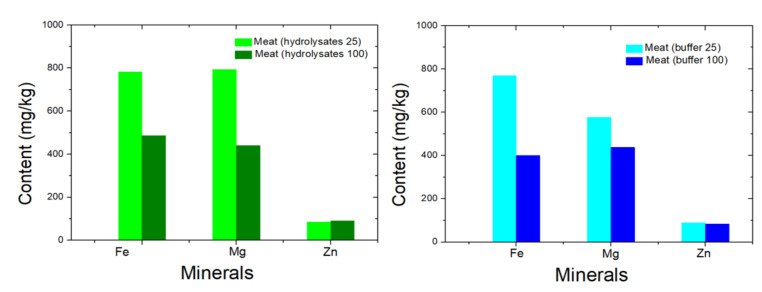
Contents of some beneficial minerals (Fe, Mg, and Zn) (mg/kg) in the intestinal digests of seal meat treated with hydrolysates (**left**) and buffer (**right**) at 25 °C and 100 °C, respectively.

Mineral analysis was performed for the (i) gastric digests and (ii) intestinal digests, but only the results of the intestinal digests are discussed in detail in this section, because the majority of minerals are absorbed in the small intestine in the human body [12]. According to previous studies on the composition of seal meat, it can be considered a good source of trace elements and vitamins. For instance, seal meat contains high amounts of important minerals, such as Fe, Zn, K, and P [10]. In this study, as shown in Table 2, the hydrolysate treatments resulted in a total Fe content of 781.99 mg/kg at 25 °C and 486.02 mg/kg at 100 °C. In the control samples treated with buffer, relatively low amounts of Fe were observed: 769.83 mg/kg (treatment at 25 °C) and 400.04 mg/kg (treatment at 100 °C). For the hydrolysate treatments, the total Mg and Zn contents were calculated as 793.99 and 84.32 mg/kg at 25 °C, respectively, and 440.31 and 90.55 mg/kg at 100 °C, respectively; for the buffer treatments, the combined Mg and Zn contents were 576.10 vs. 88.98 mg/kg at 25 °C, respectively, and 437.59 vs. 82.97 mg/kg at 100 °C, respectively. These results led to the assumption that the treatment of seal meat at 25 °C retained higher amounts of beneficial minerals in the intestinal digests than treatment at 100 °C. Moreover, the presence of seal meat hydrolysates during the treatment led to higher amounts of total Fe, Mg, and Zn in the final intestinal digests than in the buffer-treated samples. As shown in Figure 3, higher temperatures led to a general decrease in Fe, Mg, and Zn mineral contents in the digests. It is speculated that more peptides that chelate mineral ions are generated due to the higher temperature. In sum, these findings show that treatment of seal meat at 25 °C with hydrolysates is a better approach to provide and retain important minerals.

The contents of various minerals in the gastric and intestinal digests are shown in Table 2, which confirms that there were no harmful minerals, such as Pb, Mn, Mo, Ni, Hg, Sn, Ti, As, etc., present in the seal meat digests.

### 2.4. Iron Ions in Processed Seal Meat Products after Digestion

**Figure 4:**
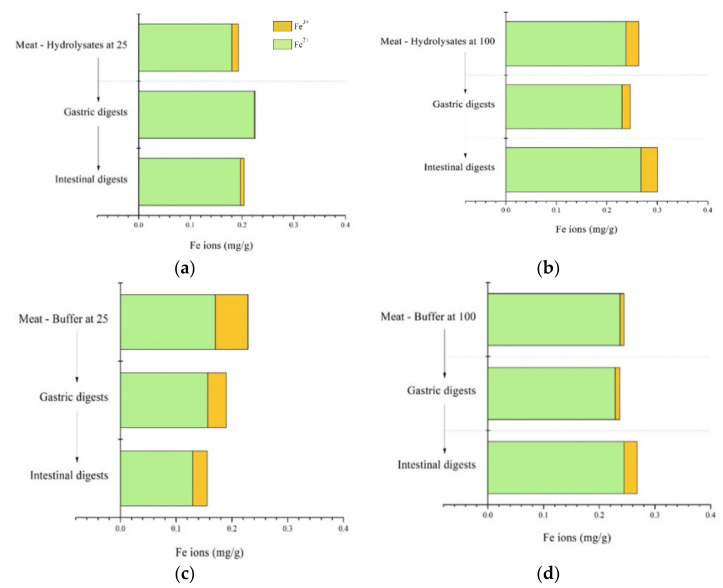
Calculated amounts of Fe^2+^ and Fe^3+^ ions (mg/g) in the gastric and intestinal digests of seal meat treated with hydrolysates at 25 °C (**a**) and 100 °C (**b**) and their control samples treated with buffer at 25 °C (**c**) and 100 °C (**d**).

The concentrations (mg/g) of Fe^2+^ and Fe^3+^ ions before and after digestion in the simulated gastric and intestinal tract were measured, and the results are presented in Figure 4 (Fe^2+^ ions in green and Fe^3+^ ions in orange). In contrast to the results regarding the Fe mineral content in Section 2.3, the hydrolysate treatment at 100 °C and buffer treatment increased the amount of free Fe ions. Seal meat treated with hydrolysates generally exhibited a higher total content of Fe ions, especially Fe^2+^, than their control counterparts treated with buffer. The antioxidant properties of the hydrolysates that were used to treat seal meat probably prevented or reduced the oxidation of Fe^2+^ to Fe^3+^, explaining the lower contents of Fe^3+^ in sample (a) compared to (c). Figure 4b,d presents the results obtained after the processing at 100 °C and shows that a higher amount of Fe^3+^ was found, which was probably due to the denaturation of antioxidant hydrolysates at elevated temperatures or the high-temperature-induced oxidation of Fe^2+^ to Fe^3+^. Fe^2+^ is of special interest for people suffering from iron deficiency, as it can be readily absorbed by the human body in the gastrointestinal tract [12]. Thus, seal meat treated with hydrolysates represents a potentially good source of iron with high bioavailability.

## 3. Methods and Materials

### 3.1. Chemicals and Biological Materials

Fresh adult harp seal meat (*Pagophilus groenlandicus*) loins were obtained from SeaDNA Canada Inc., Québec, Canada. Antioxidant hydrolysates from seal meat were prepared using enzymatic hydrolysis in phosphate buffer (pH 7.5) following an established protocol [13]. Ethylenediaminetetraacetic acid (EDTA), ethanol, 2,2-diphenyl-1-picrylhydrazyl (DPPH), iron sulfate (FeSO_4_), 1,10-phenanthroline, sodium phosphate, hydroxylammonium chloride, and hydrogen peroxide (H_2_O_2_) were purchased from Sigma-Aldrich Co. (St. Louis, MO, USA).

### 3.2. Treatment of Seal Meat with Heat and Seal Meat Hydrolysates

**Figure 5:**
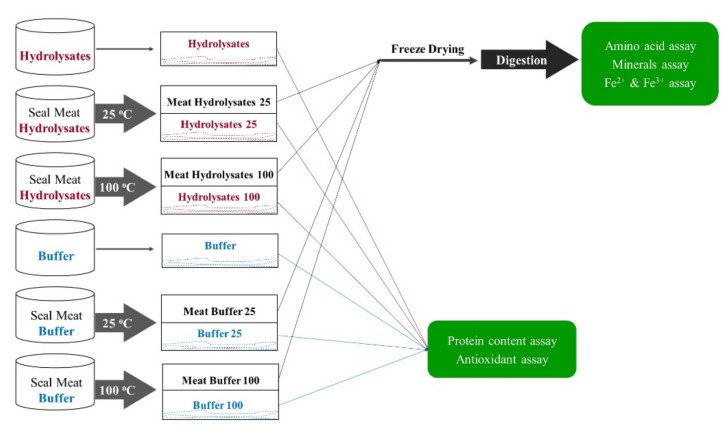
Illustration of the workflow for the treatment of seal meat with seal meat hydrolysates and buffer at 25 °C and 100 °C.

Fresh seal meat was washed and blended into minced meat before being added to the hydrolysates or the buffer solution (phosphate buffer, pH 7.5) (3:10 *w*/*v*, i.e., 3 g of meat to 10 mL of the respective solution). The treatment of seal meat in either hydrolysates or buffer solution was performed at 25 °C (representing low-temperature processing or room temperature storage conditions) and 100 °C (representing cooking conditions at high temperature) for 30 min under continuous stirring. Afterwards, the meat residues were separated from the mixture using a 0.22 µm vacuum filtration membrane. The meats treated with hydrolysates at 25 °C and 100 °C and buffer solution at 25 °C and 100 °C were labeled “Meat Hydrolysates 25”, “Meat Hydrolysates 100”, “Meat Buffer 25”, and “Meat Buffer 100”. The corresponding solutions collected after separation were labeled “Hydrolysates 25”, “Hydrolysates 100”, “Buffer 25”, and “Buffer 100”. The original “Buffer” and “Hydrolysates” solutions were utilized as controls. All samples were frozen at −40 °C and then freeze-dried to obtain a powder using a Labconco freeze-dryer before subsequent analysis. The workflow is summarized in Figure 5.

### 3.3. Determination of Antioxidant Properties

The antioxidant activity of solutions collected after separation, i.e., “Hydrolysates 25”, “Hydrolysates 100”, “Buffer 25”, and “Buffer 100”, were measured via (i) DPPH free radical scavenging activity and (ii) hydroxyl radical scavenging activity, as shown in Figure 5. Before analysis, the protein concentration of each solution was adjusted to 5 mg/mL, which was determined using a Pierce BCA protein assay kit (Thermo Scientific, Rockford, IL, USA). For the DPPH assay, the samples were mixed with a solution of DPPH in ethanol (freshly prepared), and the absorbance was measured at 517 nm using a DU 800 UV/visible spectrophotometer (Beckman Coulter Inc., Brea, CA, USA). For OH radical scavenging, the samples were mixed with 1,10-phenanthroline, FeSO_4_, and EDTA in sodium phosphate buffer (pH 7.5), followed by the addition of H_2_O_2_. The absorbance was measured at 536 nm using a spectrophotometer (Beckman, Brea, CA, USA). Both methods were performed following the protocol described in a previous study [30].

### 3.4. Digestion of Seal Meat via a Dynamic Gastrointestinal (GI) Model

The digestion of the freeze-dried forms of seal meats treated with hydrolysates at 25 °C and 100 °C and buffer solution at 25 °C and 100 °C, i.e., “Meat Hydrolysates 25”, “Meat Hydrolysates 100”, “Meat Buffer 25”, and “Meat Buffer 100” (as shown in Figure 5), was carried out using an advanced dynamic soft rat stomach model composed of the stomach, duodenum, gastric juice pump, intestinal juice pump, and the excretion tube, which was established by Pongmalai et al. [31]. In this study, the liquid digests after gastric and intestinal digestion were collected for subsequent analysis.

### 3.5. Determination of Amino Acids

The composition and contents of amino acids in gastric and intestinal digests of seal meat described in Section 3.4 (as shown in Figure 5) were measured using ACQUITY ultra-performance liquid chromatography (UPLC) system following the methods described by Zhang et al. [30,32].

### 3.6. Determination of Minerals

The composition and amounts of minerals in gastric and intestinal digests of seal meat described in Section 3.4 (as shown in Figure 5) were measured using inductively coupled plasma-mass spectroscopy (ICP-MS) (Agilent 7700 × Santa Clara, CA, USA) according to the methods reported by Lemmens et al. [33].

### 3.7. Determination of Fe Ions

The contents of Fe^2+^ and Fe^3+^ ions in gastric and intestinal digests of seal meat described in Section 3.4 (as shown in Figure 5) were measured spectrophotometrically [34]. Samples were first dissolved in distilled water, incubated for 30 min, and centrifuged at 4000 rpm for 1 min. The Fe^2+^ contents in the supernatants were measured using the 1,10-phenanthroline method. The total Fe ion content was measured using the same method after converting all Fe^3+^ to Fe^2+^ via hydroxylammonium chloride. The Fe^3+^ content was back-calculated.

### 3.8. Statistical Analyses

Triplicate measurements were performed for antioxidant activity and Fe ion determination.

## 4. Conclusions

Seal meat can be considered a good source of high-quality protein, since it contains important amino acids and minerals, especially Fe ions. In this work, seal meat was treated with seal meat hydrolysates as a preservative agent, and there was an improvement in the release and availability of beneficial amino acids and minerals. In addition, the loss of desirable Fe^2+^ ions due to the oxidation process during long-term storage and high-temperature processing was reduced due to the antioxidant properties of the hydrolysates used to treat the seal meat. Additionally, the effect of low and high temperature in combination with hydrolysate treatment showed that the digestibility and nutritional intake depended on the processing conditions. Generally, higher contents of all studied nutrients were achieved after the treatment of seal meat with hydrolysates, for which higher amounts of amino acids were found for treatment at 100 °C, whereas for mineral intake, treatment at 25 °C was shown to be the preferable processing temperature. In summary, the treatment of seal meat with seal meat hydrolysates can be considered a promising bioprocessing method to improve the biological availability and nutrient values of seal meat.

## Figures and Tables

**Table 1:** Amino acid composition (in number per 1000 amino acids) of the intestinal digests of seal meat treated with hydrolysates or buffer at 25 °C and 100 °C, respectively.

Amino Acids	Numbers (per 1000 Amino Acids)			
	Meat (Hydrolysates 25)	Meat (Hydrolysates 100)	Meat (Buffer 25)	Meat (Buffer 100)
*Non-essential amino acids (NEAA)*				
Ala	87	87	84	89
Arg	51	45	50	50
Asx (Asp+Asn)	96	92	100	88
Glx (Glu+Gln)	141	131	127	122
Gly	85	87	95	141
Pro	43	43	48	61
Ser	55	54	59	55
Tyr	24	26	27	23
*Total NEAA*	*582*	*565*	*590*	*629*
*Essential amino acids (EAA)*				
His	43	44	40	32
Ile	48	50	46	40
Leu	91	94	93	79
Lys	73	82	77	73
Met	24	22	19	19
Phe	36	41	35	35
Thr	47	45	43	41
Val	56	57	57	52
*Total EAA*	*418*	*435*	*410*	*371*
EAA/NEAA	0.718	0.770	0.695	0.590

**Table 2:** Table of the amounts of minerals (mg/kg) in gastric and intestinal digests of seal meat treated with hydrolysates and buffer at 25 °C and 100 °C, respectively.

Mineral (mg/kg)	Meat–Hydrolysates 25 °C		Meat–Hydrolysates 100 °C		Meat–Buffer 25 °C		Meat–Buffer 100 °C	
	Gastric Digests	Intestinal Digests	Gastric Digests	Intestinal Digests	Gastric Digests	Intestinal Digests	Gastric Digests	Intestinal Digests
Ag	<0.50	<0.50	<0.50	<0.50	<0.50	<0.50	<0.50	<0.50
Al	11.80	21.51	26.04	7.28	25.13	22.14	26.60	17.76
As	<0.50	<0.50	<0.50	<0.50	<0.50	<0.50	<0.50	<0.50
Au	<0.50	<0.50	<0.50	<0.50	<0.50	<0.50	<0.50	<0.50
B	<0.50	<0.50	<0.50	<0.50	<0.50	<0.50	<0.50	<0.50
Ba	<0.50	<0.50	<0.50	<0.50	<0.50	<0.50	<0.50	<0.50
Be	<0.50	<0.50	<0.50	<0.50	<0.50	<0.50	<0.50	<0.50
Bi	<0.50	<0.50	<0.50	<0.50	<0.50	<0.50	<0.50	<0.50
Ca	703.82	1024.74	768.99	1107.90	2246.46	1100.02	1206.36	750.12
Cr	36.99	60.05	48.32	40.38	63.95	65.33	44.47	64.28
Cd	<0.50	<0.50	<0.50	<0.50	<0.50	<0.50	<0.50	<0.50
Co	<0.50	<0.50	<0.50	<0.50	<0.50	<0.50	<0.50	<0.50
Cu	2.82	3.01	1.89	2.50	5.21	5.94	1.53	2.88
Fe	884.42	781.99	736.89	486.02	880.15	769.83	124.37	400.04
Ga	<0.50	<0.50	<0.50	<0.50	<0.50	<0.50	<0.50	<0.50
Ge	<0.50	<0.50	<0.50	<0.50	<0.50	<0.50	<0.50	<0.50
Hf	<0.50	<0.50	<0.50	<0.50	<0.50	<0.50	<0.50	<0.50
Hg	<0.50	<0.50	<0.50	<0.50	<0.50	<0.50	<0.50	<0.50
K	5253.20	12,547.02	4266.01	7236.46	15,027.46	11,293.84	13,885.86	13,501.73
Li	<0.50	<0.50	<0.50	<0.50	<0.50	<0.50	<0.50	<0.50
Mg	1103.74	793.99	496.35	440.31	2225.12	576.10	570.37	437.59
Mn	0.73	1.10	0.82	0.92	3.87	2.49	1.64	1.11
Mo	<0.50	<0.50	<0.50	<0.50	<0.50	<0.50	<0.50	<0.50
Na	21,647.36	77,945.86	20,824.45	50,441.43	75,538.30	61,061.62	93,029.12	91,713.28
Ni	<0.50	<0.50	<0.50	<0.50	<0.50	<0.50	<0.50	<0.50
P	6132.35	4477.88	4476.05	3592.14	15,022.37	8100.78	3771.29	3624.02
Pb	<0.50	<0.50	<0.50	<0.50	<0.50	<0.50	<0.50	<0.50
Pd	<0.50	<0.50	<0.50	<0.50	<0.50	<0.50	<0.50	<0.50
Pt	<0.50	<0.50	<0.50	<0.50	<0.50	<0.50	<0.50	<0.50
S	5491.97	4058.97	5126.93	4190.13	3856.38	6093.86	1659.23	3450.14
Sb	<0.50	<0.50	<0.50	<0.50	<0.50	<0.50	<0.50	<0.50
Se	<0.50	<0.50	<0.50	<0.50	<0.50	<0.50	<0.50	<0.50
Sn	<0.50	<0.50	<0.50	<0.50	<0.50	<0.50	<0.50	<0.50
Sr	9.75	11.63	9.77	10.46	19.27	13.23	10.57	10.97
Ti	0.69	1.11	1.46	0.76	2.94	1.63	1.69	1.72
TI	<0.50	<0.50	<0.50	<0.50	<0.50	<0.50	<0.50	<0.50
V	<0.50	<0.50	<0.50	<0.50	<0.50	<0.50	<0.50	<0.50
W	<0.50	<0.50	<0.50	<0.50	<0.50	<0.50	<0.50	<0.50
Zn	72.02	84.32	66.41	90.55	102.23	88.98	67.77	82.97
Zr	<0.50	<0.50	<0.50	<0.50	<0.50	<0.50	<0.50	<0.50
